# Supplementary material for: Comparison of journal and top publisher self-citation rates in COVID-19 research
Source: PLoS One. 2024 Dec 5;19(12):e0314976. doi: 10.1371/journal.pone.0314976 (PMC11620575; doi:10.1371/journal.pone.0314976)
Supplement: S1 File — (DOCX) [file pone.0314976.s001.docx]

***Supplementary file 1.***

Search strategy on COVID-19 research in WoS

| **N°** | **Steps** |
| --- | --- |
| **1** | **Combination of TITLE or KEY** |
|  | *TI = ( "2019-nCoV" OR "COVID-19" OR "coronavirus disease 2019" OR "SARSCoV- 2" OR "HCoV-2019" OR "hcov" OR "NCOVID-19" OR "severe acute respiratory syndrome coronavirus 2" OR "severe acute respiratory syndrome corona virus 2" OR "SARS-CoV2" OR covid2019 OR "COVID-19" OR covid19 OR 2019ncov OR "2019 ncov" OR covid19 OR "covid 19" OR "sars cov 2" OR sars2 ) OR AK = ( "2019-nCoV" OR "COVID-19" OR "coronavirus disease 2019" OR "SARS-CoV-2" OR "HCoV-2019" OR "hcov" OR "NCOVID-19" OR "severe acute respiratory syndrome coronavirus 2" OR "severe acute respiratory syndrome corona virus 2" OR "SARS-CoV2" OR covid2019 OR "COVID-19" OR covid19 OR 2019ncov OR "2019 ncov" OR covid19 OR "covid 19" OR "sars cov 2" OR sars2 )* |
| **2** | **Constraints** **related to document types** |
|  | Article, early access, letter, review article, and editorial material |
| **3** | **Constraints related to publications years** |
|  | 2020, 2021, 2022, 2023 |

Search strategy on Tuberculosis research in WoS

| **N°** | **Steps** |
| --- | --- |
| **1** | **Combination of TITLE or KEY** |
|  | *TI = ( tuberculo* OR ( ( tb AND disease ) OR ( tb AND infect* ) ) ) OR TI = ( "koch* disease" OR "pott* disease" OR scrofula* OR mantoux ) OR AK = ( tuberculo* OR ( ( tb AND disease ) OR ( tb AND infect* ) ) ) OR AK = ( "koch* disease" OR "pott* disease" OR scrofula* OR mantoux )* |
| **2** | **Constraints** **related to document types** |
|  | Article, early access, letter, review article, and editorial material |
| **3** | **Constraints related to publications years** |
|  | 2020, 2021, 2022, 2023 |

Search strategy on HIV/AIDS research in WoS

| **N°** | **Steps** |
| --- | --- |
| **1** | **Combination of TITLE or KEY** |
|  | *TI = ( "HIV-1" OR "Human Immunodeficiency Virus" OR "HIV infect*" OR "HIV test*" OR "HIV prevent*" OR "Acquired Immunodeficiency Virus" OR "Acquired Immunodeficiency Syndrome" OR "HIV/AIDS" OR "HIV-AIDS" ) OR TI = ( hiv AND ( "antiretroviral" OR haart OR "integrase inhibitor*" OR "reverse transcriptase inhibitor*" ) ) OR AK = ( "HIV-1" OR "Human Immunodeficiency Virus" OR "HIV infect*" OR "HIV test*" OR "HIV prevent*" OR "Acquired Immunodeficiency Virus" OR "Acquired Immunodeficiency Syndrome" OR "HIV/AIDS" OR "HIV-AIDS" ) OR AK = ( hiv AND ( "antiretroviral" OR haart OR "integrase inhibitor*" OR "reverse transcriptase inhibitor*" ) )* |
| **2** | **Constraints** **related to document types** |
|  | Article, early access, letter, review article, and editorial material |
| **3** | **Constraints related to publications years** |
|  | 2020, 2021, 2022, 2023 |
